# Sentiment analysis in public health: a systematic review of the current state, challenges, and future directions

**DOI:** 10.3389/fpubh.2025.1609749

**Published:** 2025-06-20

**Authors:** Ismael Villanueva-Miranda, Yang Xie, Guanghua Xiao

**Affiliations:** ^1^Department of Health Data Science and Biostatistics, University of Texas Southwestern Medical Center, Dallas, TX, United States; ^2^Department of Bioinformatics, University of Texas Southwestern Medical Center, Dallas, TX, United States

**Keywords:** sentiment analysis, natural language processing, mental health, LLM, public health, systematic review

## Abstract

**Introduction:**

Sentiment analysis, using natural language processing to understand opinions in text, is increasingly relevant for public health given the volume of online health discussions. Effectively using this approach requires understanding its methods, applications, and limitations. This systematic review provides a comprehensive overview of sentiment analysis in public health, examining methodologies, applications, data sources, challenges, evaluation practices, and ethical considerations.

**Methods:**

We conducted a systematic review following PRISMA guidelines, searching academic databases through Semantic Scholar and screening studies for relevance. A total of 83 papers analyzing the use of sentiment analysis in public health contexts were included.

**Results:**

The review identified a trend toward the use of advanced deep learning methods and large language models (LLMs) for a wide range of public health applications. However, challenges remain, particularly related to interpretability and resource demands. Social media is the predominant data source, which raises concerns about data quality, bias, linguistic complexity, and ethical issues.

**Discussion:**

Sentiment analysis offers the potential for gaining public health insights but faces significant methodological, data-related, and ethical challenges. Reliable and ethical application demands rigorous validation, improved model interpretability, the development of ethical frameworks, and continued research to support responsible development and deployment.

## 1 Introduction

Understanding public perspectives is important to effective public health practice, particularly in addressing health crises, developing policies, and designing communication strategies (1, 2). In recent years, the rapid expansion of digital communication channels, especially social media platforms, has created large, real-time repositories of public opinions, emotions, and experiences related to health (3, 4). As a result, this user-generated text offers exceptional opportunities for public health intelligence, enabling large-scale monitoring and potentially more responsive interventions (5, 6).

In this context, sentiment analysis (SA), also known as opinion mining, has emerged as an important computational tool within natural language processing (NLP) to systematically identify, extract, and analyze subjective information from text (3). Its application in public health is growing, with researchers using SA to measure public reactions to health policies (2), monitor population mental health signals (7), enhance infectious disease surveillance (8, 9), understand patient experiences (10), and identify communication challenges such as health misinformation (11, 12).

However, applying SA effectively and responsibly in this domain involves many complexities. In particular, researchers must select from diverse methodologies, ranging from lexicon-based approaches to traditional machine learning and advanced deep learning models, including the rapidly growing LLMs field (13–15). Each method offers distinct advantages and limitations involving accuracy, interpretability, data requirements, and computational costs. In addition, the main data sources–especially social media platforms like Twitter–present challenges related to data quality, noise, representativeness, and ethical concerns regarding privacy, consent, and bias (8, 11, 16). Although SA has significant promise, translating its findings into meaningful public health impact requires careful methodological choices, rigorous evaluation, and responsible implementation (1, 12).

Given the expanding use and evolving nature of SA in public health, a systematic synthesis of current practices, challenges, and outcomes is needed. Therefore, this review aims to provide a comprehensive overview of the landscape of sentiment analysis applications within the public health domain, focusing on studies published between 2020 and 2025. Specifically, we seek to address the following research questions:

(RQ1) What are the predominant sentiment analysis methodologies and evaluation metrics currently employed in sentiment analysis in public health research?
(RQ2) What types of data sources are most commonly utilized for sentiment analysis in public health, and what are the primary ethical considerations discussed in relation to this data collection and analysis?
(RQ3) How are LLMs being utilized for sentiment analysis tasks within the public health context, and what are the reported advantages or challenges compared to other methods?
(RQ4) How have sentiment analysis findings been used to inform or influence public health interventions, communication strategies, or policy-making processes?

By addressing these questions, this review intends to map the current state of the field, identify key methodological and ethical considerations, evaluate the emerging role of LLMs, and assess the evidence for the practical impact of sentiment analysis in advancing public health goals.

## 2 Methods

This systematic review adhered to the Preferred Reporting Items for Systematic Reviews and Meta-Analyses (PRISMA) guidelines (17) to ensure a transparent and reproducible methodology. The search strategy, detailed below, was configured to retrieve studies relevant to the primary research questions concerning sentiment analysis in public health, as presented in the Introduction.

### 2.1 Search strategy

**Figure 1:**
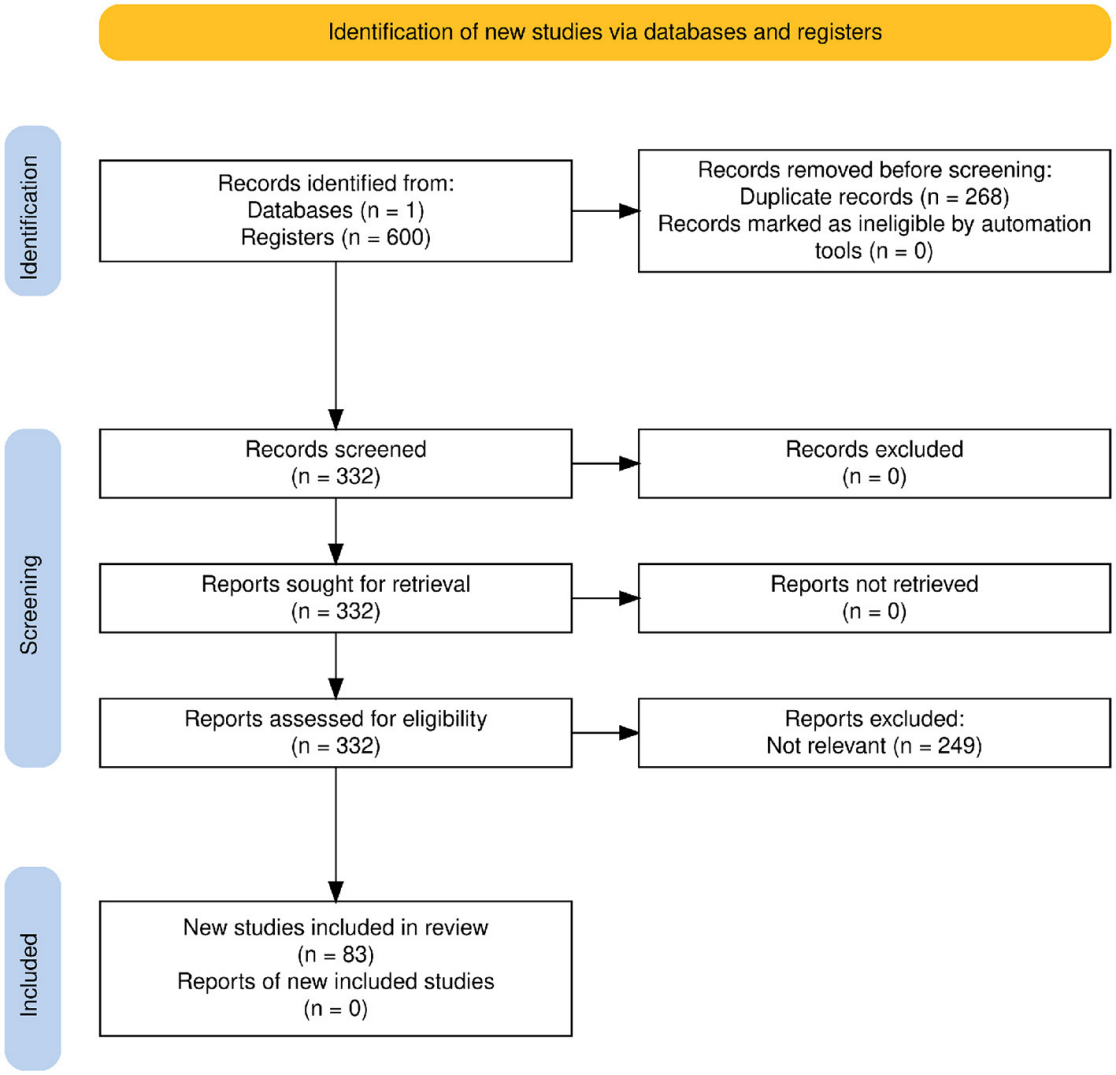
PRISMA 2020 flow diagram illustrating the study identification, screening, eligibility, and inclusion process.

The systematic search was conducted exclusively using Semantic Scholar (18), as indicated in the PRISMA diagram (Figure 1, *n* = 1 database). Semantic Scholar was chosen as the only database due to its extensive coverage, indexing nearly 200 million papers from a multitude of prominent publishers and repositories, including PubMed, Springer Nature, ACM, IEEE, and arXiv, among others. Its AI-powered search capabilities and comprehensive indexing across various scientific disciplines, including medicine, public health, and computer science, provided a robust foundation for identifying relevant literature for this review.

The review started by defining four core research areas to capture the breadth of relevant literature pertinent to sentiment analysis in public health: (1) Sentiment Analysis Methods in Public Health; (2) Data Sources, Preprocessing, and Ethical Considerations in Public Health Sentiment Analysis; (3) the application of LLMs in Public Health Sentiment Analysis; and (4) the Impact of Sentiment Analysis on Public Health Interventions and Policy Making. These areas were chosen to cover the key methodologies, data handling aspects, emerging technologies, and practical applications in this field.

Search queries were developed based on these identified research areas. For each area, three types of queries were formulated: a broad query for comprehensive scope, a focused query using the “+” operator for higher specificity, and a related query incorporating alternative or supplementary terms using the “|” operator. This multi-search strategy, executed within Semantic Scholar as previously detailed, was designed to balance the retrieval of a broad scope of relevant studies with the precise identification of highly relevant results. Table 1 presents the complete set of search queries.

**Table 1:** Search topics and query strategies for the systematic review.

**Search topic**	**Broad query**	**Focused query**	**Related query**
Sentiment Analysis Methods in Public Health	“Sentiment Analysis Methods in Public Health”	“Sentiment Analysis” + “Public Health” + “Methods”	“Natural Language Processing in Healthcare” | “Emotion Detection in Health Communication” | “Text Mining in Epidemiology”
Data Sources, Preprocessing, and Ethical Considerations in Public Health Sentiment Analysis	“Data Sources Preprocessing Ethical Considerations Public Health Sentiment Analysis”	“Data Sources” + “Preprocessing” + “Ethical Considerations” + “Public Health” + “Sentiment Analysis”	“Machine Learning in Public Health” | “Natural Language Processing in Healthcare” | “Ethics in AI for Health”
Large Language Models (LLMs) in Public Health Sentiment Analysis	“Large Language Models Public Health Sentiment Analysis”	“Large Language Models” + “Public Health” + “Sentiment Analysis”	“Natural Language Processing in Healthcare” | “AI in Public Health” | “Sentiment Analysis in Healthcare”
Impact of Sentiment Analysis on Public Health Interventions and Policy Making	“Sentiment Analysis Public Health Interventions Policy Making”	“Sentiment Analysis” + “Public Health” + “Policy Making”	“Natural Language Processing Public Health Decision Making”

### 2.2 Inclusion and exclusion criteria

To ensure the relevance and focus of this review, specific inclusion and exclusion criteria were applied during the study selection process. Studies were included if they directly addressed aspects of sentiment analysis within the public health domain, aligning with one or more of the four core research areas detailed in Section 2.1 (Search Strategy).

Specifically, “public health relevance” was determined by assessing if a study's primary content and objectives involved the application, methodological development, or critical discussion of sentiment analysis in contexts directly pertinent to population health. These contexts included, but were not limited to:

- Monitoring, surveillance, or prediction of diseases and health conditions (e.g., infectious disease outbreaks, chronic diseases, mental health trends).
- Evaluation of public health interventions, policies, or communication campaigns (e.g., vaccine programs, anti-smoking campaigns, responses to health crises).
- Understanding patient experiences, healthcare quality, or access to health services from a population perspective.
- Analyzing public discourse, attitudes, or behaviors related to health topics (e.g., health-related misinformation, substance use, health equity).

For a study to be included, it had to demonstrate a clear and primary connection to public health goals, applications, or outcomes, rather than focusing solely on clinical informatics for individual patient care without broader public health implications, or general NLP methods without a specific public health application of sentiment analysis. No specific keyword thresholds, beyond the initial search query terms, were applied during the screening or full-text eligibility assessment for determining relevance; instead, relevance was judged thematically based on the study's main research questions, methods, and discussion in relation to our defined research areas and the public health contexts listed above.

Only journal articles, conference papers, or studies published in English from January 2020 to March 2025 were considered. Studies were excluded if they were:

- Editorials, commentaries, or letters only.
- Not primarily focused on sentiment analysis within a public health context. For example, studies were excluded if sentiment analysis was applied to purely commercial product reviews without a health link, or if they were general natural language processing studies on clinical text (e.g., EHR analysis) that did not incorporate a sentiment analysis component for broader public health insights.
- Not published in English.

Following these criteria, a total of 249 reports were excluded during the full-text assessment stage, primarily because their core focus was not aligned with the specific research scope of this review concerning sentiment analysis in public health (see Figure 1).

### 2.3 Study selection process

The initial search retrieved 600 records from the selected database. After removing 268 duplicate records during the identification phase, 332 unique records remained for screening.

All 332 records were screened based on their titles and abstracts. No records were excluded at this stage. The full texts of all 332 potentially relevant records were retrieved and assessed for eligibility according to the inclusion and exclusion criteria described in Section 2.2. During the full-text assessment, 249 reports were excluded, primarily because they did not meet the relevance criteria for the review's focus. This selection process resulted in 83 studies being included in the final qualitative synthesis, as shown in the PRISMA flow diagram (Figure 1).

## 3 Results

### 3.1 Methods for analyzing health sentiments

**Figure 2:**
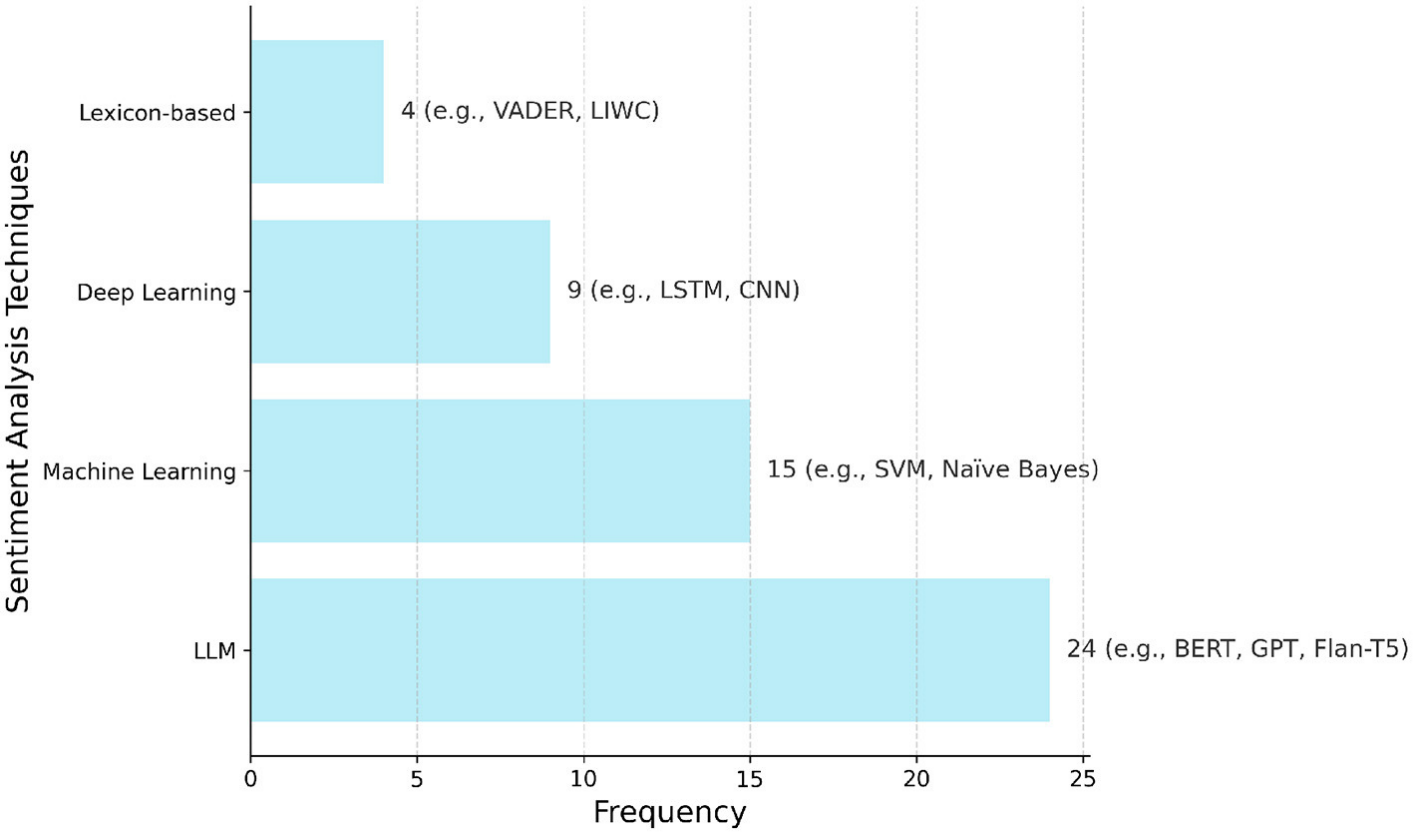
Frequency of the main sentiment analysis techniques reported in the 83 studies included in this review. Techniques are grouped into four categories: large language models (LLMs), machine learning (ML), deep learning (DL), and lexicon-based methods. Bars indicate the number of studies that applied each category, with example models/techniques shown alongside the corresponding frequency values (e.g., BERT, Naïve Bayes, LSTM, VADER).

This section addresses the first research question (RQ1) by reviewing the principal sentiment analysis methodologies, including lexicon-based approaches, traditional machine learning, deep learning, LLMs, and hybrid methods, as summarized in Table 2. As illustrated in Figure 2, there is a notable distribution in the application of these techniques across the studies included in this review, with Large Language Models (LLMs) being the most frequently reported category. A summary of the key characteristics of the 83 studies included in this review, detailing study types, primary sentiment analysis methods used, data sources analyzed, and public health application areas, is presented in Table 3.

**Table 2:** Comparison of sentiment analysis methodologies for public health data.

**Methodology**	**Principle**	**Strengths**	**Weaknesses**	**Public health use cases**	**Example tools and algorithms**
Lexicon-based	Uses predefined dictionaries (lexicons) with word sentiment scores to calculate overall text sentiment.	Simple, interpretable, computationally inexpensive, no large labeled training data required.	Struggles with context, sarcasm, negation, domain-specific language; performance depends heavily on lexicon quality/coverage.	Quick broad assessments, resource-limited settings. Risky for complex health topics without domain adaptation/validation. Often performs poorly off-the-shelf.	VADER, SentiWordNet, LIWC
Traditional machine learning	Trains classifiers (e.g., SVM, NB) on labeled data to predict sentiment in new text using engineered features.	Learns patterns beyond keywords, can capture more context than lexicons if trained well.	Requires substantial labeled training data (costly/time-consuming in health); domain-dependent performance; may struggle with complex language.	Good baseline when domain-specific labeled data is available. Suitable for many classification tasks but often outperformed by DL.	SVM, Naive Bayes (NB), Logistic Regression (LR), Random Forest (RF)
Deep learning (RNN/LSTM)	Uses recurrent neural networks to model sequential information and context in text.	Effective at capturing sequential dependencies and context.	Can be computationally intensive; may be surpassed by Transformers for complex context.	Suitable for analyzing sequential text data like social media posts or patient narratives where context flow is important.	LSTM, GRU
Deep learning (transformers/LLMs)	Uses attention mechanisms (Transformers) or massive pre-training (LLMs) for deep language understanding.	State-of-the-art performance, excellent contextual understanding; fine-tuning reduces data needs; LLMs offer zero/few-shot potential.	Computationally expensive, can be “black boxes”; LLM performance on health data inconsistent without tuning/prompting.	Preferred for high accuracy on complex tasks. Fine-tuning often needed for PH. LLMs promising for rapid analysis but require careful validation.	BERT, RoBERTa, GPT, XLNet, T5, ChatGPT
Hybrid models	Combines elements from lexicon, traditional ML, and/or DL approaches.	Potential to leverage strengths of multiple methods, improve robustness/accuracy, incorporate domain knowledge.	Can increase complexity; effectiveness depends on the specific combination and task.	Promising for integrating domain knowledge (e.g., medical lexicons) with data-driven models. May offer balance between performance and interpretability. Needs more PH-specific exploration.	Lexicon features + ML; Lexicon annotation + DL; CNN+LSTM

**Table 3:** Summary of studies applying sentiment analysis in public health contexts.

**References**	**SA method(s) mentioned**	**Data source(s)**	**Public health application area**
Zhang et al. (16)	Qualitative analysis (implicit)	Social media, Internet Comm.	Research ethics
Takats et al. (8)	Sentiment mining, surveillance	Twitter	Research ethics, methodology
Simmering and Huoviala (37)	LLM (GPT-4, GPT-3.5), ABSA	SemEval-2014 Task 4	Method development (ABSA)
Marshall et al. (7)	NLP Platform (Commercial)	Twitter (UK)	Mental health surveillance
Zhang et al. (34)	NLP (hierarchical transformer)	Sina Weibo	Mental health (depression pred.)
Idaikkadar et al. (65)	Data science (general)	Internet data (implied)	Research ethics (injury prev.)
Pandey et al. (63)	Classifier (WEKA), Python speech	NHS reviews (web scraping)	Patient experience/healthcare qual.
Nandy and Dubey (74)	NLP (framework)	Written DHI data	Mental health, interventions
Aliyuda (6)	Health analytics (ML, Stats)	Various health data	Public health crises response
Chen et al. (24)	ML (LR, NB, RF), topic modeling	Weibo	Patient experience
Shang et al. (84)	Event analysis, regression	Stock prices, investor sentiment	Public health emergencies (Econ.)
Wolfenden et al. (70)	Survey analysis	Policy-maker/Practitioner Survey	Health policy/intervention eval.
Nayak and Raghatate (2)	NLP-ISA (integrated SA)	Public discourse	Health policy/delivery eval.
Nadel and Smith (82)	Data science (mining images)	Public records (Emails)	Public health crises (Gov. Resp.)
Prasinos et al. (1)	Big data analytics, ontology	Heterogeneous health data	Health policy making (hearing loss)
Gordon (85)	Policy analysis	Wellbeing indicators	Health policy making (wellbeing)
Li et al. (40)	LLM (ChatGPT), ABSA, ICD-11 API	Patient reviews	Healthcare resource allocation
Chen et al. (79)	SEM, factor analysis	Survey data (China)	Health policy compliance
Adenyi et al. (5)	Big data analytics (ML, Pred Mod)	Heterogeneous health data	Public health decision-making
Gille et al. (83)	Policy research	Not specified	Health policy making (trust)
Sajadi et al. (71)	Mixed methods (review, dialogues)	Literature, expert interviews	Health policy making (motivation)
Ansah et al. (69)	Scoping review	Literature (Africa)	Health policy making (politics)
Zhu et al. (35)	LLM (Llama-3), SMLM (XLM-R)	Cross-lingual SA datasets	Method development (cross-lingual)
Shaikh et al. (51)	LLM (ChatGPT), DL	Student feedback dataset	Education (sentiment analysis)
Zhang et al. (36)	LLM, SLM	Multiple SA datasets	Method evaluation (LLM vs SLM)
Arias et al. (3)	Sentiment analysis (general)	Social media	Mental health (COVID-19)
Gandy et al. (19)	NLP (VADER, T2D, LIWC), LLM(ChatGPT)	YouTube comments	Method evaluation, opioid epidemic
Wang et al. (72)	Deep learning, multimodal fusion	Multimodal data (implied)	Method development (multimodal)
Alzaidi et al. (29)	Deep learning (text-inception)	Drug experience datasets	Patient experience (Drugs)
Deng et al. (68)	LLM, Semi-supervised Learning	Reddit	Market sentiment analysis
Albladi et al. (13)	NLP (ML, DL, hybrid), LLM	Twitter	Method review (Twitter SA)
Maehlum et al. (43)	LLM (Norwegian), Human annotation	Patient surveys (Norway)	Patient experience, method eval.
Pan and Xu (30)	Deep Learning (BERT_RCNN)	Weibo	Public health emergencies
Elmitwalli et al. (42)	LLM (Flan-T5), LoRA Fine-tuning	Twitter (Tobacco/E-cig)	Health Comm. (Tobacco Control)
Sharma and Sindhu (67)	NLP (linguistic, emotional models)	Audio data	Method development (Audio SA)
Bouaraki et al. (66)	LLM (BERT), sentiment analysis	Not specified (fake news data)	Misinformation detection
Mao et al. (38)	PLM (Prompt-based)	Sentiment/emotion datasets	Method evaluation (PLM bias)
Molenaar et al. (20)	NLP (VADER), topic modeling (LDA)	Twitter (Australia)	Food security
Mughal et al. (33)	Deep learning, LLM (PaLM, GPT)	ABSA datasets	Method comparison (ABSA)
Wang et al. (52)	LLM (explanation generation)	ABSA datasets	Method development (ABSA Bias)
Sheng et al. (27)	Deep learning (CNN, RNN, LSTM)	Healthcare network public opinion	Public opinion monitoring
Mohammad (11)	Conceptual analysis	AER/SA literature	Research ethics (AER/SA)
Challapalli (28)	Deep learning (CNN, LSTM, BiLSTM)	Twitter	Method development (Twitter SA)
Naous et al. (54)	LLM (Arabic/Multilingual)	CAMeL dataset (Reddit-derived?)	Method evaluation (Cultural Bias)
Kanungo (9)	NLP (ML, DL)	Social media	Crisis management
Niu et al. (56)	Event study, sentiment analysis	Twitter (Canada)	Policy evaluation (COVID NPIs)
Russell et al. (12)	Machine learning SA	Twitter (Jamaica)	Policy evaluation (COVID Restr.)
Correia et al. (73)	SA (XLM-RoBERTa), Topic Mod(BERTopic)	Twitter	Public perception (obesity)
Khandelwal et al. (53)	Neurosymbolic (NN + knowledge)	Twitter, Reddit, News	Mental health surveillance(COVID)
Putri et al. (21)	Naïve Bayes, Lexicon-based	Instagram	Mental health (depression)
Marques et al. (22)	NLP (VADER), Stats	Online news (Brazil)	Health campaign Eval. (Syphilis)
Hu (23)	SnowNLP, correlation analysis	Sina Weibo	Public health emergencies (COVID)
Keyworth et al. (81)	Survey comparison, regression	Healthcare professional surveys	Health interventions (behavior Ch.)
White et al. (32)	SA (Fine-tuned DistilRoBERTa)	Twitter	Health communication (COVID/Vax)
Yu et al. (60)	SnowNLP	Weibo (Wuhan)	Public emotions (COVID Outbreak)
Aldosery et al. (31)	RNN, embedded topic model	Twitter (UK)	Policy response (COVID-19)
Wang et al. (64)	NLP (LDA, BERT), human analysis	Twitter (US Cities)	Health communication (COVID/Vax)
Watkins et al. (61)	Discourse & Sentiment analysis	Facebook Group (UK)	Health communication (COVID-19)
Asthana et al. (80)	Scoping review	Literature	Governance (COVID-19 Decision)
Brall et al. (77)	Qualitative interviews	Policy makers, scientists (Swiss)	Ethics in policy making (COVID)
Khalaf et al. (25)	NLP (TF-IDF), ML (LSTM, SVM etc.)	Twitter	Vaccine sentiment (COVID-19)
Muhtar et al. (57)	Mixed methods (Content & SA)	Social media	Health advocacy/awareness
Cheng et al. (58)	Sentiment analysis, Correlation	Twitter (England)	Vaccine sentiment/uptake (COVID)
Abrams et al. (78)	NLP, sentiment analysis	Twitter, Facebook (Legislators)	Mental health (HCW Burnout)
Bulut and Poth (59)	Sentiment analysis, stats	Public health briefings (Canada)	Health communication (COVID-19)
Yigitcanlar et al. (4)	Social media analytics (Geo-Twitter)	Twitter (Australia)	Policy decisions (COVID-19)
Lee et al. (46)	LLM (GPT-4) vs. human clinicians	Telemental health intake data	Mental health (crisis prediction)
Espinosa and Salathe (15)	LLM (GPT), Rule-based SA	Social media (vaccination)	Method Evaluation (LLM Stance)
Yang et al. (41)	LLM (ChatGPT), Prompting	Mental health datasets	Mental health analysis (XAI)
Ma et al. (62)	Qualitative interviews	Mental health/AI experts (China)	Mental health practice (LLM Integ)
Ferrario et al. (55)	Critical analysis	Literature (Philosophy, Psych)	Mental health (LLM ethics/robust.)
Jo et al. (49)	Focus groups, interviews	Chatbot users/operators/Devs	Public health intervention (LLM)
De Angelis et al. (14)	Perspective/analysis	AI/LLM literature	Infodemic/misinformation (LLMs)
Chen et al. (39)	LLM (DoT prompting)	Not specified (therapy context)	Mental health (therapy training)
Fang et al. (50)	LLM, Statistical analysis	Wearable data (Fitbit)	Personalized health insights
Sood et al. (45)	LLM (GPT-3.5, BERT), ML	Student voice survey	Mental health support (college)
Wu et al. (47)	LLM (Data augmentation - CALLM)	Clinical interview transcripts	Mental health diagnosis (PTSD)
Bauer et al. (44)	LLM (sentence embedding), Dim. Red.	Reddit (Mental Health subs)	Mental health (suicidality lang.)
Wang et al. (48)	LLM (Patient Simulation PATIENT-Ψ)	Not specified (therapy context)	Mental health (therapy training)
Gatto et al. (75)	NER (EP S-BERT)	HealthE Dataset (health advice)	Method development (Health NER)
Cardoso et al. (26)	NLP (Tokenization), ML (NN, XGB)	CONITEC Reports (Brazil)	Health technology assessment (HTA)
Polisena et al. (10)	Lexicon-based, ML	Social media	Health technology assessment (HTA)
Vij and Prashant (76)	ML (CSVM-DTO), NLP (Word2Vec)	EHR data	Public health decision (EHR Sem.)

#### 3.1.1 Lexicon-based methods

Lexicon-based methods provide a foundational approach to sentiment analysis. They rely on predefined dictionaries, or lexicons, in which words are assigned sentiment scores indicating polarity and sometimes intensity. Well-known examples include VADER and LIWC (19). The overall sentiment of a text is typically calculated by aggregating the scores of its individual words, with some methods adjusting for linguistic features such as negation or intensification. As shown in Figure 2, these lexicon-based approaches were applied in 4 of the reviewed studies.

Compared to machine learning approaches, lexicon-based methods are valued for their simplicity, interpretability, and lower computational demands. They do not require large labeled training datasets, making them practical for initial analyses or in settings with limited resources. In public health research, they have been used to analyze social media discussions on food security (20) and mental health (3, 21), as well as to assess sentiment in health-related news articles (22).

However, lexicon-based methods face significant limitations. They often struggle to capture context-dependent sentiment, including sarcasm or irony. Their performance depends heavily on the quality, coverage, and domain relevance of the chosen lexicon (10). General-purpose lexicons often perform poorly in specialized fields like public health, where word meanings can differ (e.g., a “positive” test result) and specialized terminology may be absent or misclassified. Moreover, analyzing noisy texts from sources such as social media requires continuous updates to lexicons, which can be costly and time-consuming (23). Indeed, studies comparing tools like VADER and LIWC against manual coding for health topics on social media have found only fair levels of agreement (19).

Therefore, the practicality of standard lexicon-based methods is limited for many public health applications, especially those requiring a deeper understanding of complex discussions (10). Achieving reliable results often requires the development or adaptation of domain-specific lexicons. In addition, using these methods involves ethical considerations related to data handling and interpretation (11). While helpful in providing broad overviews, the inherent limitations of lexicon-based methods must be carefully weighed against the specific goals of the public health analysis (13).

#### 3.1.2 Traditional machine learning methods

Traditional machine learning (ML) provides a data-driven alternative to lexicon-based rules for sentiment analysis. Figure 2 shows that these traditional ML methods were reported in 15 of the included studies. These methods typically use supervised learning, where an algorithm learns from text data previously labeled with sentiment categories (e.g., positive, negative, neutral) (3, 13). The trained model identifies patterns linking text features to sentiment, allowing it to classify new, unlabeled text.

Several standard ML algorithms are commonly used for sentiment analysis in health-related research. These include Naïve Bayes (NB), Support Vector Machines (SVM), Logistic Regression (LR), Random Forests (RF), and others (24–26). SVMs, in particular, have often been a popular choice in health sentiment analysis tasks (10, 23). For example, NB has been applied to classify sentiment regarding depression based on Instagram comments (21), while combinations of LR, NB, and RF have been used to analyze patient experience themes on Weibo (24). Various ML algorithms, including SVM and RF, have also been compared to analyze COVID-19 vaccine sentiment on Twitter (25) and predict health technology assessment outcomes (26).

An important step in these ML approaches is feature engineering, which converts raw text into numerical formats that algorithms can process. Common techniques include Bag-of-Words (BoW) and Term Frequency-Inverse Document Frequency (TF-IDF) (25). TF-IDF weights words based on their importance within a document relative to a larger collection of documents, helping to prioritize more distinctive terms (25). N-grams, which are sequences of adjacent words, can also capture some local word order information.

The main strength of traditional ML methods is their ability to learn complex patterns from data beyond simple word matching, capturing more context than basic lexicons if trained on relevant data (9). They can perform well when enough high-quality labeled data specific to the health domain is available.

However, their primary weakness is this dependence on labeled training data. Creating such datasets for specialized fields like public health can be costly and time-consuming, requiring domain expertise. Model performance is often domain-dependent; a model trained on one text type (like product reviews) may not perform well on another (like patient forum posts). While sometimes better than lexicons, traditional ML models can still struggle with complex language features like sarcasm or implied meanings and may show lower accuracy compared to deep learning models, especially on complex tasks or noisy data like microblogs (23). Therefore, while traditional ML methods offer a solid baseline for public health sentiment analysis when domain-specific data is available, their data requirements and potential limitations in language understanding must be considered.

#### 3.1.3 Deep learning methods

Deep Learning (DL) models use artificial neural networks with multiple layers to automatically learn complex patterns from text data, often reducing the need for manual feature engineering. According to Figure 2, deep learning techniques, excluding LLMs which are reported separately, were utilized in nine studies.

Early successful approaches included Recurrent Neural Networks (RNNs) and variants like Long Short-Term Memory (LSTM) networks (25, 27). These models process text sequentially, allowing them to capture word order and context dependencies (27). For example, LSTMs have been used effectively for analyzing sentiment in Twitter data regarding COVID-19 vaccines (25) and compared with other DL models like CNNs and BiLSTMs (28). Convolutional Neural Networks (CNNs), known primarily for image analysis, have also been adapted to identify local patterns in text (27). Hybrid models combining CNNs and RNNs (like Bi-GRU or LSTM) have also been developed (29) and applied, for instance, to analyze sentiment during public health emergencies (30) or assess drug experiences (29). RNNs have also been combined with topic modeling for analyzing public reactions to the COVID-19 pandemic on Twitter (31).

Recently, Transformer-based architectures have become highly effective in natural language processing (13). Models like BERT (Bidirectional Encoder Representations from Transformers) and its variants (e.g., RoBERTa, DistilRoBERTa, DeBERTa) are pre-trained on large amounts of text, enabling them to develop a deep understanding of language (13). These pre-trained models can then be fine-tuned for specific tasks, often achieving high performance (32, 33). Transformers have been applied in various health contexts, such as predicting depression risk from social media posts (34) or analyzing COVID-19 vaccine sentiment (32). Models like BERT have formed the basis for more complex architectures like BERT-RCNN, designed to improve sentiment classification accuracy for public health emergency texts compared to traditional ML and simpler DL models (30).

The strength of DL models lies in their ability to automatically learn complex features and contextual relationships from data, leading to strong performance on complex language tasks. Fine-tuning pre-trained Transformer models can also reduce the need for extensive labeled data compared to training traditional ML models from scratch. However, DL models can be computationally intensive to train and operate, and their complex internal workings can make them challenging to interpret (acting as “black boxes”) (30). Their performance still depends on the quality and relevance of the training or fine-tuning data. For public health, DL models, particularly fine-tuned Transformers, are increasingly favored when high accuracy and detailed understanding are needed, provided computational resources and relevant data are available.

#### 3.1.4 Large language models (LLMs)

Addressing the third research question (RQ3), this subsection explores how LLMs are being specifically utilized for sentiment analysis tasks within public health, detailing their reported advantages and challenges compared to other methods.

Addressing the third research question (RQ3), this subsection explores how LLMs are being specifically utilized for sentiment analysis tasks within public health, detailing their reported advantages and challenges compared to other methods. As showed in Figure 2, LLMs were the most frequently employed category of sentiment analysis techniques, appearing in 24 of the reviewed studies. LLMs, such as the GPT series (e.g., GPT-3.5, GPT-4), Flan-T5, PaLM, and Llama, represent the latest evolution in deep learning for language (35, 36). These models are pre-trained on extremely large and diverse text datasets, giving them remarkable abilities to understand and generate human-like text (14). For sentiment analysis, LLMs offer flexible approaches like zero-shot learning (performing tasks without specific examples) or few-shot learning (learning from a small number of examples) (15, 35, 36). This is particularly relevant when labeled data is scarce (36). Techniques like prompt engineering (designing detailed instructions) (37–39) and chain-of-thought prompting (40) can influence LLM performance in zero-shot and few-shot settings (37, 41). LLMs can also be fine-tuned on domain-specific data, often achieving state-of-the-art results (37, 42).

LLMs are being explored across various public health and medical domains. Applications include analyzing patient feedback on healthcare (40, 43), understanding public discourse on topics like vaccination (15) or tobacco (42), analyzing mental health discussions on social media (41, 44, 45), predicting mental health crises (46), detecting cognitive distortions in psychotherapy (39), augmenting clinical data (47), simulating patients for training (48), supporting public health interventions via chatbots (49), and generating personalized health insights from wearable data (50). Comparative studies show fine-tuned LLMs like GPT-3.5 or Flan-T5 can outperform previous methods in specific tasks like aspect-based sentiment analysis (33, 37) or student feedback analysis (51), though performance varies depending on the task complexity and model (33, 36). Some studies suggest LLMs can approach clinician-level performance in specific prediction tasks (46) or generate high-quality explanations for mental health analysis (41, 52).

However, using LLMs presents challenges. While powerful, their performance can be inconsistent, especially on specialized health data without careful fine-tuning or prompting (19, 36, 43). Comparisons show they may lag behind specialized models in complex tasks (36) or be outperformed by other approaches in specific contexts (53). They are computationally expensive (37) and often lack interpretability (41). Significant ethical concerns exist regarding their potential to generate misinformation (contributing to an “AI-driven infodemic”) (14), perpetuate biases present in their training data (e.g., cultural or demographic biases) (38, 46, 54), and issues related to humanization and robustness when used in sensitive applications like mental health support (49, 55). Rigorous validation, attention to bias mitigation, and careful consideration of ethical guidelines are necessary before deploying LLMs for critical public health tasks (15, 19, 41).

#### 3.1.5 Hybrid models

Hybrid approaches in sentiment analysis aim to leverage the strengths of different methodologies–such as lexicon-based, traditional machine learning (ML), and deep learning (DL) models–to potentially improve performance or balance accuracy with interpretability (13). Various combinations exist. For instance, lexicon-derived sentiment scores can serve as features for ML models, or ML techniques can help refine domain-specific lexicons. Within DL, architectural hybrids like combining Convolutional Neural Networks (CNNs) and Long Short-Term Memory (LSTM) networks have been explored. Another strategy uses lexicon methods for initial data annotation, which then helps train or fine-tune ML or DL classifiers. Neurosymbolic methods that integrate neural networks with symbolic knowledge sources like lexicons also represent a hybrid approach, adapting dynamically to evolving language in domains like mental health (53). Furthermore, insights from one type of model, like explanations generated by LLMs, can be used to enhance the performance and reduce spurious correlations in other sentiment analysis models (52).

The main benefit of hybrid models lies in synergizing rule-based knowledge (from lexicons) with data-driven pattern recognition (from ML/DL), potentially leading to more robust and accurate sentiment classification (29). Some studies have reported superior results using hybrid methods compared to single approaches alone, for example, when analyzing medicine reviews.

In the public health context, hybrid strategies offer a promising way to integrate valuable domain knowledge, such as medical terminology or health-specific sentiment lexicons, into powerful data-driven models. This could produce accurate and better-adapted models to the specific language of health-related text. Such approaches might offer a better balance between prediction performance and the ability to understand the reasoning behind sentiment classifications, which is often important for actionable public health insights. However, the effectiveness of specific hybrid combinations needs further investigation and validation across diverse public health applications.

### 3.2 Applications of sentiment analysis across the public health

To address the fourth research question (RQ4), this section reviews how sentiment analysis findings have been applied across various public health domains, examining the evidence for their use in informing or influencing interventions, communication strategies, or policy-making processes.

#### 3.2.1 Monitoring public response to health policies and campaigns

Sentiment analysis provides a valuable tool for monitoring public responses to health policies and communication campaigns. Authorities can use it to track public opinion toward specific initiatives, such as healthcare reforms, COVID-19 mitigation measures (like lockdowns or mask mandates), vaccination programs, or tobacco control regulations, often in near real-time and at a large scale (2, 4, 12). This allows for a dynamic understanding of public acceptance or resistance, offering advantages over traditional polling methods (4). Studies have used sentiment analysis on social media data, like Twitter, to measure public reactions to COVID-19 restrictions (12) or non-pharmaceutical interventions (56), sometimes finding links between negative sentiment and lower policy compliance (12) or positive sentiment shifts following intervention announcements (56). Sentiment analysis can also help evaluate the effectiveness of public health campaigns, for example, those promoting disease awareness like syphilis (22) or discouraging tobacco use (42). By analyzing sentiment shifts or content engagement related to campaign activities, officials can gain insights into message effectiveness (22, 57). For instance, analyzing online news revealed that syphilis testing increased more significantly in response to campaign messages designed to induce attitude change compared to purely informational ones (22). Similarly, tracking sentiment related to COVID-19 vaccines has shown correlations between positive online sentiment and vaccine uptake rates, particularly among certain demographics (58). Sentiment analysis can also be applied to assess the consistency and reception of official public health communications, such as briefings during a crisis (59). Identifying specific aspects of policies or campaigns that provoke strong reactions allows health communicators to address public concerns and refine their strategies, eventually informing better policy decisions (2, 4, 12).

#### 3.2.2 Enhancing infectious disease surveillance and outbreak detection

Sentiment analysis can potentially complement traditional methods for infectious disease surveillance (8). By monitoring large volumes of text from sources like social media, it may help detect outbreaks or changes in disease activity earlier than conventional systems (6). This involves tracking mentions of symptoms (e.g., “fever,” “cough”) combined with negative sentiment, which might signal actual illness reports. Systems sometimes referred to as “Social Media Epidemic Intelligence” aim to identify unusual clusters of negative health-related sentiment that could indicate an emerging public health event.

Beyond early detection, sentiment analysis is valuable for tracking public awareness, concerns, and emotional responses during ongoing epidemics, such as COVID-19 (23, 31, 60). Analyzing the volume and sentiment of discussions provides insights into the public's psychological state, including levels of fear or anxiety, and helps identify specific concerns at different stages of an event (60). For instance, studies analyzing Weibo posts during the COVID-19 pandemic in China observed distinct emotional trajectories across outbreak stages, with negative emotions often triggered by specific milestone events like the confirmation of human-to-human transmission (23, 60). Understanding these dynamics can help health authorities adapt their communication and support efforts more effectively (61). Some research has also explored using sentiment data within epidemiological models to predict disease spread dynamics better.

#### 3.2.3 Monitoring mental health signals and population wellbeing

Analyzing sentiment and emotional expression in online text offers a way to monitor mental health trends at the population level (3). User-generated content from social media platforms like Twitter, Weibo, Reddit, or Instagram, as well as specialized online health forums, can be analyzed to detect shifts in collective mood or increases in expressions related to depression, anxiety, or stress (7, 21, 34, 53). This approach recognizes that language use often reflects subjective well-being (7). For example, studies have used natural language processing (NLP) to analyze tweets about mental health during the COVID-19 pandemic (7, 53) or to predict depression risk based on language patterns observed on platforms like Weibo (34).

A particularly sensitive application involves using NLP and sentiment analysis to identify individuals potentially at risk of suicide by analyzing their online posts (44). Research using large language model (LLM) based techniques on platforms like Reddit has explored linguistic dimensions associated with suicidality, identifying themes such as disconnection, burdensomeness, and hopelessness (44). This application, particularly concerning suicide risk prediction using online data (44), is ethically complex (raising considerations of privacy and potential harm, as discussed broadly in Section 3.5.1) but highlights the potential of computational methods, including LLMs (11, 46). LLMs are also being explored for other mental health applications, such as analyzing clinical data (47), detecting cognitive distortions (39), simulating patients for training (48), understanding student mental health support needs (45), and potentially augmenting therapeutic interactions (41). However, as detailed in our discussion of LLM methodologies (Section 3.1.4), challenges concerning their robustness, potential for bias (a critical ethical concern further explored in Section 3.5.3), and appropriate humanization in sensitive interactions remain significant considerations (55, 62).

Sentiment analysis has also been applied within online support groups focused on mental health or addiction. Analyzing discussions in these communities can help researchers understand patient experiences, identify unmet needs, and examine the dynamics of peer support. Furthermore, monitoring sentiment related to subjective well-being itself can offer broader insights into population mental states beyond specific diagnoses.

#### 3.2.4 Understanding patient experiences and healthcare quality

Sentiment analysis is increasingly applied to understand patient perspectives on healthcare services, treatments, and overall experiences. Researchers can mine patient feedback from online sources like hospital or physician review sites (e.g., RateMDs, WebMD, NHS reviews), general consumer platforms (e.g., Yelp, Google), social media, and patient forums (10, 24, 63). Analyzing these reviews helps measure patient satisfaction, identify specific areas needing improvement in healthcare quality, and classify provider performance (24, 63). For example, analyzing patient experience posts on Weibo helped identify key discussion themes like healthcare professionals' attitudes and access to care (24).

Beyond general satisfaction, sentiment analysis can explore patient experiences with specific treatments. By analyzing discussions about pharmaceuticals, vaccines, or therapies, researchers assess real-world patient perceptions of effectiveness, side effects, and value (10, 29). Such analysis of online drug reviews may help identify unreported adverse drug reactions contributing to pharmacovigilance (29). LLMs are also being explored to analyze patient feedback. For instance, ChatGPT has been used for aspect-based sentiment analysis of patient reviews to assess dissatisfaction with different aspects of care, like physician skills or infrastructure (40). Other studies evaluate LLMs for annotating sentiment in free-text patient survey comments (43). There is also emerging interest in applying sentiment analysis to patient-provider communications or clinical notes, although privacy remains a key consideration. The goal is often to gain actionable insights that can lead to better quality of care and improved patient outcomes (63).

#### 3.2.5 Navigating health communication and combating misinformation

Sentiment analysis helps understand how health information and misinformation spread and are received within the complex modern information environment (9). It can be used to analyze how public sentiment shapes message dissemination and how sentiment itself is influenced by information exposure, including from influential figures or during specific events (32). Understanding public discourse is important for effective health communication, especially during crises (59, 64).

An important application is identifying health-related misinformation, rumors, or stigmatizing language (11). Detecting clusters of negative sentiment, unusual discussion patterns, or narratives associated with false information (e.g., about vaccines, disease origins, or treatments like tobacco/e-cigarettes) can alert public health authorities to emerging threats (15, 42). This capability of LLMs to generate convincing text (see Section 3.1.4) means they could also accelerate the spread of misinformation at an unprecedented scale, creating an “AI-driven infodemic” (14).

Insights from sentiment analysis–understanding the specific concerns, fears, or beliefs driving negative sentiment–can inform adapted public health communication strategies (2, 64). By addressing the root causes of negative sentiment and providing clear, targeted information, authorities can work to build trust, improve risk communication, and counter misinformation (57, 61). Analyzing public discussions, for example on social media platforms regarding sensitive topics like the opioid epidemic, can provide valuable feedback for communication efforts (19). Understanding how the public receives messages is key, as negative reception might correlate with lower compliance with health recommendations (12).

### 3.3 Data sources for public health sentiment analysis

Focusing on the second research question (RQ2), this section details the types of data sources, such as social media, health forums, news media, and patient reviews, most commonly utilized for sentiment analysis in public health.

**Figure 3:**
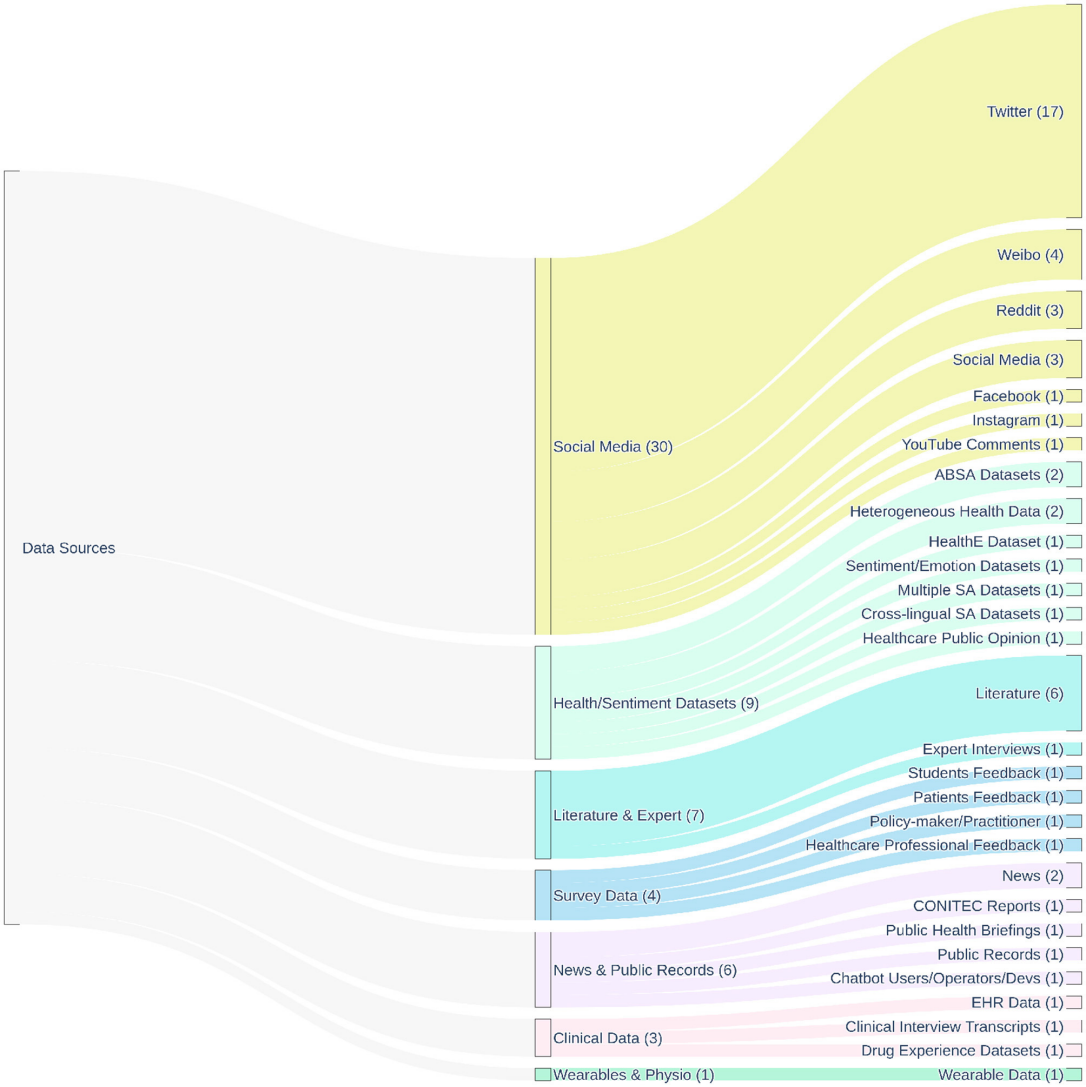
Hierarchical distribution of the main data sources used across the 83 studies included in this review, all of which applied sentiment analysis in public health. The diagram illustrates the relationship between broad data source categories (e.g., Social Media, Survey Data, Clinical Data) and their specific instances (e.g., Twitter, Reddit, EHR data). Flow widths are proportional to the number of studies referencing each source. The numbers in parentheses next to each node indicate the frequency of use across the included studies, highlighting the predominance of social media platforms–particularly Twitter—as primary input data for sentiment analysis.

Focusing on the second research question (RQ2), this section details the types of data sources, such as social media, health forums, news media, and patient reviews, most commonly utilized for sentiment analysis in public health. Figure 3 provides a hierarchical overview of the main data sources identified in the reviewed studies, illustrating the relationships between broad data categories and specific instances, and highlighting the predominance of social media platforms.

#### 3.3.1 Leveraging social media: opportunities and caveats

Social media platforms are currently the most common data source for public health sentiment analysis (8), a trend clearly visible in Figure 3, where they collectively represent the largest category of data sources (30 instances across various platforms). Twitter (now X) has been particularly prevalent in research, also highlighted in Figure 3 as the most frequent individual platform (17 instances), largely due to its accessible API and public nature (8, 13). However, researchers also utilize other platforms like Facebook (61), Reddit (44, 53), Weibo (23, 24, 34), Instagram (21), and YouTube (19), each having unique user demographics and communication styles. Many studies across diverse health topics such as COVID-19 responses (4, 12, 31, 56), mental health (7), vaccination (15, 58), and tobacco use (42) rely on these platforms.

The main opportunity social media offers is access to large amounts of real-time data reflecting spontaneous public opinions and reactions (4, 9). This allows for timely identification of emerging health trends and public concerns, often at a lower cost than traditional survey methods.

However, using social media data presents significant challenges, or caveats. The data is often noisy and unstructured, featuring informal language, slang, misspellings, emojis, and hashtags that complicate NLP tasks (23, 28). Robust data preprocessing is necessary but complex. A major limitation is representativeness; social media users do not accurately reflect the general population, with biases related to age, socioeconomic status, location, and other factors (8, 65). Therefore, findings derived exclusively from social media may lack generalizability, potentially underrepresenting vulnerable groups (8). Furthermore, significant limitations include issues of data representativeness and demographic biases, as well as the impact of platform policy volatility on research access and continuity. These critical aspects are discussed in detail as major research challenges in Section 3.4.2 Privacy and ethical considerations are important (8, 16, 65). Even public data requires careful handling regarding consent, anonymity, and the risk of re-identification (16). The reliability of information is also a concern, as misinformation, spam, and automated bots can distort sentiment trends (8). The potential for LLMs to rapidly generate content could further exacerbate this issue (14). Finally, platform data access policies can change, impacting research continuity (8).

#### 3.3.2 Mining online health communities and forums

Specialized online health communities and patient forums represent another potential data source for sentiment analysis. These include platforms dedicated to specific conditions or general health discussions, as well as health-focused groups or subreddits on broader social media platforms like Facebook or Reddit (44, 53, 61).

The main advantage of these sources is the potential to access richer, more detailed narratives about patient experiences, symptoms, and treatment journeys compared to typical short posts on social media. Analyzing discussions within these communities, such as mental health subreddits (44, 53) or support groups (61), can provide deep insights into the lived experiences and unmet needs of individuals with specific health conditions. For example, analyzing language in suicidality-focused subreddits using Large Language Model (LLM) techniques helped identify key emotional themes expressed by users (44).

However, several caveats apply. Data volumes are usually much smaller than on large social media platforms. Participants often represent a self-selected group, which may introduce biases related to health status or motivation to seek online support. Privacy considerations are often amplified due to the sensitive nature of the health information shared (16). Gaining access to data from these platforms can be challenging, and many datasets are not publicly available due to ethical constraints (16). Researchers must carefully consider the ethical implications of analyzing conversations within these communities, ensuring user privacy and avoiding intrusion (16).

#### 3.3.3 Analyzing news media and digital publications

Online news articles, blogs, and other digital publications represent another category of text data suitable for sentiment analysis. Analyzing sentiment in these sources can help researchers understand media framing of health issues, track public discourse influenced by news coverage, and assess sentiment shifts related to major events or communications (2, 22). Sentiment analysis of news and digital publications has been used, for example, to evaluate public health campaigns by examining media coverage during the campaign period (22) or to understand discourse around health policy reforms (2). News articles were also included alongside social media data in analyses of mental health sentiment during the COVID-19 pandemic (53).

A primary caveat when analyzing news media is that the expressed sentiment often reflects journalistic perspectives or organizational messaging rather than direct public opinion. Therefore, analysis must carefully distinguish between the sentiment inherent in the events reported and the sentiment conveyed by the author or publication. Furthermore, the volume of relevant articles for specific health topics may be lower compared to user-generated content on social media. The prevalence of fake news or misinformation disseminated through news-like digital formats also presents a challenge, requiring careful source evaluation; techniques combining LLMs and sentiment analysis are being explored for fake news detection (66).

#### 3.3.4 Utilizing patient reviews and survey responses

Direct patient feedback, collected through online review platforms or surveys, offers a targeted data source for sentiment analysis. This includes reviews of healthcare providers, clinics, hospitals, or specific products like pharmaceuticals posted on dedicated sites (e.g., RateMDs, NHS Choices) or general consumer platforms (e.g., Google Reviews, Yelp) (29, 63). Open-ended text responses within patient surveys also provide rich qualitative data suitable for sentiment analysis (43, 45).

The primary opportunity lies in obtaining specific feedback about particular services or experiences, which can yield detailed insights valuable for quality improvement, patient safety monitoring, and understanding satisfaction (10, 63). Analyzing patient reviews, whether using machine learning or LLMs, can help identify dissatisfaction with specific aspects of care, such as physician skills or administration (40), or assess sentiment regarding drug efficacy and side effects (29). Sentiment analysis of patient survey comments is also an area where LLMs are being evaluated (43, 45). Furthermore, survey data may sometimes be linked to demographic information, allowing for more stratified analysis.

However, patient reviews can suffer from bias, as individuals with very strong positive or negative opinions may be more likely to post feedback, potentially skewing the overall sentiment picture. Collecting survey data can be more resource-intensive than mining publicly available online data. Additionally, both reviews and survey responses often contain sensitive personal health information, requiring strict data handling protocols to ensure privacy and ethical compliance.

### 3.4 Challenges in public health sentiment analysis

While sentiment analysis offers considerable potential for providing valuable public health insights, significant challenges often restrict its practical and reliable application. These limitations span the entire analysis process, from inherent linguistic complexities and data limitations to ethical considerations and evaluation difficulties. This section examines the main obstacles encountered when applying sentiment analysis within the public health domain, discussing issues related to language complexities, data quality and bias, resource constraints, ethical responsibilities, and the rigorous validation of findings.

#### 3.4.1 Deciphering linguistic complexities: context, sarcasm, and health jargon

Human language presents significant challenges for automated sentiment analysis due to its complexity and context-dependence. The sentiment polarity of words can change dramatically based on surrounding text or the broader situation (67). For instance, the word “positive” typically indicates favorable sentiment, but a “positive” test result in healthcare carries negative implications for the patient. Automated tools may struggle to capture these context shifts (19). Rhetorical devices like sarcasm and irony, which invert literal meaning, are notoriously difficult for algorithms to interpret correctly (67). Sentiment can also be expressed implicitly, requiring deeper semantic understanding.

The health domain introduces unique linguistic challenges. It possesses a specialized vocabulary, including medical jargon, acronyms, and informal patient terms (e.g., “brain fog”) that general-purpose NLP tools may not understand (29). The specific language used by individuals discussing certain conditions, like depression, may also differ in systematic ways (34). Furthermore, the inherently negative meaning of many symptom words (e.g., “pain”) can bias sentiment results toward negativity, even when the text's purpose is neutral reporting. The dynamic nature of language, especially on social media platforms with evolving slang and abbreviations, further complicates analysis (23, 53).

Even advanced LLMs, despite their capabilities outlined in Section 3.1.4, face specific challenges with linguistic complexity in health contexts. Ensuring their robustness requires considering the specific language patterns of different patient groups or cultural contexts (54, 55). The way prompts are formulated and label words are chosen can also significantly influence how LLMs interpret sentiment, highlighting their sensitivity to linguistic framing (38). While LLMs can analyze complex language (44), effective sentiment analysis in health often requires domain adaptation to handle these specific linguistic characteristics (29).

#### 3.4.2 Addressing data quality, representativeness, and bias

The reliability of sentiment analysis results is significantly affected by the quality and characteristics of the underlying data. Unstructured text, particularly from social media, often contains “noise” such as errors, inconsistent formatting, irrelevant content, spam, and bot-generated posts (5, 23). Addressing this requires robust data cleaning and preprocessing methods (13, 28).

A fundamental challenge, especially with social media data, is representativeness (8). Users of online platforms do not accurately reflect the general population, exhibiting biases related to demographics like age, socioeconomic status, geographic location, and digital literacy (5, 8). Consequently, findings based exclusively on such data may not be generalizable, and vulnerable populations might be underrepresented (8). Content bias can also occur, as individuals may selectively share information or performatively express opinions online.

A fundamental challenge, especially with social media data from predominant platforms like Twitter, is its inherent lack of representativeness (8). Users of these platforms do not accurately mirror the general population, exhibiting significant biases related to demographics such as age, socioeconomic status, geographic location (e.g., urban vs. rural), educational attainment, and digital literacy (5, 8). For instance, Twitter users often trend younger and are more concentrated in urban areas compared to the broader population. Therefore, findings derived exclusively from such data may lack generalizability and can lead to a deformed understanding of population-wide sentiment. Public sentiment surveillance might underestimate issues prevalent in older, less digitally connected, or rural populations, while public opinion on health policies could be biased if more vocal or digitally active groups are overrepresented. Failure to account for these demographic biases can inadvertently lead to interventions that do not serve all populations equitably, potentially widening existing health disparities. Vulnerable populations, in particular, might be systematically underrepresented in these digital conversations (8). Content bias can also occur, as individuals may selectively share information or performatively express opinions online, further complicating the interpretation of sentiment as truly representative public opinion.

Beyond data representativeness, a critical operational challenge arises from the volatility of platform policies and data access, particularly concerning dominant commercial platforms like Twitter (now X). These platforms are privately controlled entities whose terms of service, API access (including functionality and cost), data retention policies, and even core platform features can change abruptly and with limited notice to the research community (8). Such platform policy volatility poses considerable risks to public health research and surveillance efforts. For example, sudden restrictions or increased costs for API access can halt ongoing research projects, prevent the replication of previous studies (thereby restricting scientific verification), and make longitudinal analyses of health trends over time exceptionally difficult or impossible. Furthermore, the discontinuation of specific data streams or features can render previously effective sentiment analysis models or data collection strategies obsolete. This instability makes sustained, reliable public health monitoring based solely on these sources precarious and underscores the need for researchers and public health agencies to consider data source diversification and contingency planning.

Furthermore, algorithmic bias presents a significant risk to the validity and fairness of sentiment analysis findings (11, 65). All sentiment analysis models, from traditional machine learning to LLMs, can inherit and even amplify biases present in their training data or underlying lexicons (46, 65). For instance, LLMs have shown susceptibility to biases related to cultural contexts or linguistic framing (38, 54). Such biases can lead to skewed insights and potentially inequitable public health outcomes. A comprehensive discussion of algorithmic bias as an ethical concern, including its manifestations and mitigation strategies, is provided in Section 3.5.3. Therefore, addressing data quality and representativeness is intrinsically linked to ethical considerations of fairness (16, 19, 65). Evaluating models for fairness and mitigating bias are therefore important steps (65), alongside acknowledging data limitations and ethical considerations (16, 19).

#### 3.4.3 Addressing data scarcity and resource limitations in health contexts

Obtaining high-quality, relevant data for developing and validating sentiment analysis models in health remains a significant challenge. Large-scale, publicly available annotated datasets designed to diverse health topics are scarce. This scarcity arises from the high cost and effort required for expert annotation (43), the complexity of health language, and stringent privacy regulations (like HIPAA) that limit the sharing of sensitive patient information (16). This lack of labeled data delays the development and evaluation of supervised machine learning (ML) and deep learning (DL) models. Similarly, comprehensive, publicly accessible sentiment lexicons designed specifically for the health domain are lacking, and developing them is resource-intensive (30).

The advent of LLMs offers potential ways to mitigate some data scarcity issues. LLMs can perform reasonably well in few-shot learning scenarios, reducing the need for extensive labeled datasets (15, 36). Techniques like semi-supervised learning using LLMs (68) or using LLMs for data augmentation (47) are being explored to overcome data limitations, potentially at lower costs than traditional data collection (47). LLMs are also being evaluated for automated annotation tasks (43).

However, resource limitations extend beyond data. As noted in the discussion of LLM methodologies (Section 3.1.4), advanced DL models, particularly LLMs, demand significant computational resources (e.g., GPUs, processing time) for training and deployment, posing a substantial barrier in many public health settings (30, 37). Some approaches may offer lower computational demands than others (53). Implementing and managing these complex models also requires specialized technical expertise, which may not be available in all public health settings.

Finally, language barriers pose another challenge. Most existing sentiment analysis tools, datasets, and research focus heavily on English (35). Developing and validating methods for other languages, especially low-resource languages or those with distinct cultural contexts, requires significant effort and resources (35, 54).

#### 3.4.4 Challenges in evaluating model performance and validity

Evaluating the performance and validity of sentiment analysis models carefully is a critical but often overlooked task in public health research. Weak evaluation can damage trust in these tools and limit their usefulness (8). Poor or shallow validation can lead to unreliable models, misleading public health findings, wrong policy decisions, and eventually a decline in trust in sentiment analysis for public health.

**Figure 4:**
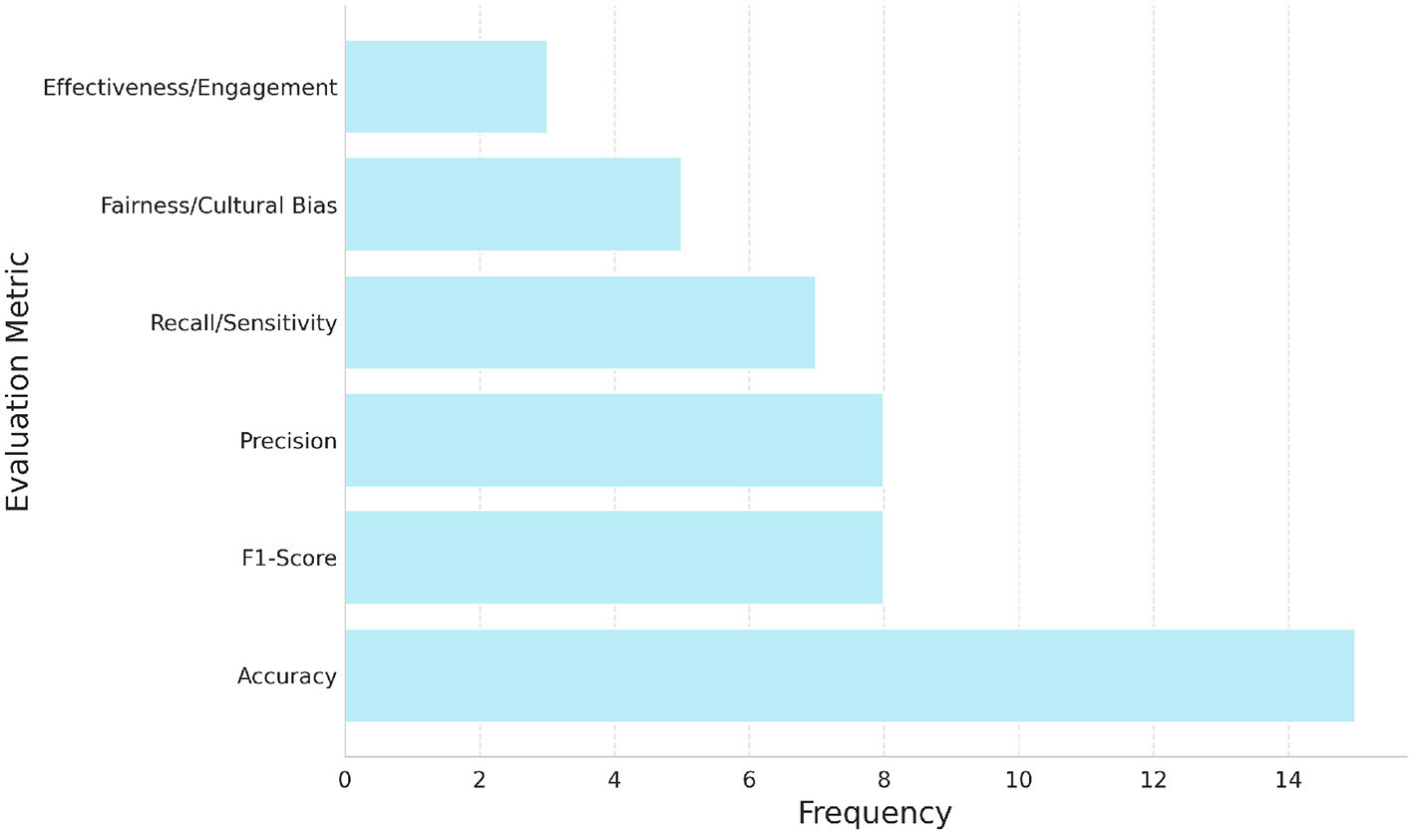
Frequency of main six evaluation metric categories reported across the 83 studies included in this review. Accuracy was the most commonly reported metric, followed by F1-score, precision, and recall. A smaller number of studies incorporated fairness- or engagement-related metrics, reflecting an emerging emphasis on ethical and participatory evaluation frameworks in public health sentiment analysis.

A main concern found in the literature is the overuse of standard metrics without enough understanding of when and how they should be used. Metrics like accuracy, precision, recall, and F1-score are often reported (24–26, 34). Our review (Figure 4) also shows that accuracy is the most common metric. However, this practice can be risky. Relying mainly on accuracy, especially when using imbalanced datasets (for example, when certain health concerns are rare), can hide poor model performance on smaller classes and create a false sense of success (19). Better metrics for imbalanced data, such as weighted F1-score (34) and the Matthews Correlation Coefficient (MCC) (19), are available, but they are not always used or explained clearly.

Many studies also confuse internal validation (testing on part of the same dataset) with readiness for real-world use. There is often a lack of external validation using independent datasets that differ by time, population, data source, or specific health topic. This is important because good results on benchmark datasets often do not mean good results on new, real-world data (36). Models must be systematically tested for generalization and robustness using diverse and realistic datasets (8). Our review shows that although some studies recognize this need, true external validation is still are.

Because sentiment is subjective, strong human benchmarking is needed, but this also reveals many inconsistencies. Comparing model outputs to human judgments with metrics like Cohen's Kappa (19) is necessary. However, the quality and transparency of the gold-standard datasets used are often not strong enough. Studies comparing different sentiment analysis tools (lexicon-based, ML, DL, LLMs) on the same health data often find large differences and only fair to moderate agreement with human annotators (19, 43). This shows that tool choice can strongly affect results and that creating high-quality annotations for health data is very difficult.

There is also a serious lack of standard practices for reporting how models are evaluated. This makes it hard to reproduce results, compare studies, or build better methods (8). Missing or unclear information about datasets (such as class imbalance), preprocessing steps, model settings, or evaluation methods creates barriers to understanding and progress.

Therefore, sentiment analysis in public health research needs a strong shift toward more complete and critical evaluation practices. Researchers must go beyond simply reporting a few metrics on limited datasets. Strong evaluations should include: (1) detailed dataset descriptions (size, source, time, preprocessing, possible biases, and class distributions), (2) a clear explanation of why certain evaluation metrics are used and how class imbalance is handled, (3) careful human benchmarking with clear annotation guidelines and agreement measurements like Cohen's Kappa, (4) strong evidence of external validation using multiple, diverse datasets, and (5) deep error analysis to explain not just how often models fail but why they fail (37). Where possible, evaluations should also include tests in real-world or simulated public health environments (19, 36).

Adopting these careful and transparent validation methods is not only a good practice but a necessary step to ensure that sentiment analysis tools are truly reliable, useful, and ethical for public health.

#### 3.4.5 Addressing data scarcity and resource limitations in health contexts

Obtaining high-quality and relevant data for developing and validating sentiment analysis models in health is a major challenge. Large-scale, publicly available datasets annotated for different health topics are rare. This problem exists because expert annotation is expensive and time-consuming (43). The complexity of health-related language and strict privacy laws, such as HIPAA, also make it difficult to share sensitive patient information (16). Without enough labeled data, it is harder to develop and test supervised machine learning (ML) and deep learning (DL) models. In addition, there are few comprehensive, publicly available sentiment lexicons designed for the health domain, and creating them requires significant resources (30).

The rise of large language models (LLMs) offers some ways to reduce data scarcity problems. LLMs can work well in few-shot learning scenarios, which lowers the need for large labeled datasets (15, 36). Researchers are also exploring techniques like semi-supervised learning with LLMs (68) and data augmentation (47) to overcome data limitations, often at lower costs than traditional methods (47). LLMs are also being tested for automatic annotation tasks (43).

However, resource limitations are not only about data. As discussed in Section 3.1.4, advanced DL models, especially LLMs, require a lot of computing power (e.g., GPUs and long processing times) for training and deployment. This creates major barriers for many public health institutions (30, 37). Some approaches need fewer computational resources than others (53). Managing these complex models also needs specialized technical skills, which are not always available in public health settings.

Language barriers are another important issue. Most sentiment analysis tools, datasets, and research focus mainly on English (35). Developing and testing methods for other languages, especially low-resource languages or languages from different cultural backgrounds, requires extra effort and resources (35, 54).

#### 3.4.6 Challenges in evaluating model performance and validity

Evaluating the performance and validity of sentiment analysis models carefully is a critical but often overlooked task in public health research. Weak evaluation can damage trust in these tools and limit their usefulness (8). Poor or shallow validation can lead to unreliable models, misleading public health findings, wrong policy decisions, and eventually a decline in trust in sentiment analysis for public health.

A main concern found in the literature is the overuse of standard metrics without enough understanding of when and how they should be used. Metrics like accuracy, precision, recall, and F1-score are often reported (24–26, 34). Our review (Figure 4) also shows that accuracy is the most common metric. However, this practice can be risky. Relying mainly on accuracy, especially when using imbalanced datasets (for example, when certain health concerns are rare), can hide poor model performance on smaller classes and create a false sense of success (19). Better metrics for imbalanced data, such as weighted F1-score (34) and the Matthews Correlation Coefficient (MCC) (19), are available, but they are not always used or explained clearly.

Many studies also confuse internal validation (testing on part of the same dataset) with readiness for real-world use. There is often a lack of external validation using independent datasets that differ by time, population, data source, or specific health topic. This is important because satisfactory results on benchmark datasets often do not mean good results on new, real-world data (36). Models must be systematically tested for generalization and robustness using diverse and realistic datasets (8). Our review shows that although some studies recognize this need, true external validation is still rare.

Because sentiment is subjective, strong human benchmarking is needed, but this also reveals many inconsistencies. Comparing model outputs to human judgments with metrics like Cohen's Kappa (19) is necessary. However, the quality and transparency of the gold-standard datasets used are often not strong enough. Studies comparing different sentiment analysis tools (lexicon-based, ML, DL, LLMs) on the same health data often find large differences and only fair to moderate agreement with human annotators (19, 43). This shows that tool choice can strongly affect results and that creating high-quality annotations for health data is very difficult.

There is also a serious lack of standard practices for reporting how models are evaluated. This makes it difficult to reproduce results, compare studies, or build better methods (8). Missing or unclear information about datasets (such as class imbalance), preprocessing steps, model settings, or evaluation methods creates barriers to understanding and progress.

Therefore, sentiment analysis in public health research needs a strong shift toward more complete and critical evaluation practices. Researchers must go beyond simply reporting a few metrics on limited datasets. Strong evaluations should include: (1) detailed dataset descriptions (size, source, time, preprocessing, possible biases, and class distributions), (2) a clear explanation of why certain evaluation metrics are used and how class imbalance is handled, (3) careful human benchmarking with clear annotation guidelines and agreement measurements like Cohen's Kappa, (4) strong evidence of external validation using multiple, diverse datasets, and (5) deep error analysis to explain not just how often models fail but why they fail (37). Where possible, evaluations should also include tests in real-world or simulated public health environments (19, 36).

Adopting these careful and transparent validation methods is not only a good practice but a necessary step to ensure that sentiment analysis tools are truly reliable, useful, and ethical for sentiment analysis in public health.

### 3.5 Ethical considerations in mining public health sentiments

Further addressing the second research question (RQ2), this section discusses the primary ethical considerations reported in relation to data collection, analysis, and application of sentiment analysis in public health contexts, summarized in Table 4.

**Table 4:** Ethical challenges and mitigation strategies in sentiment analysis for public health.

**Ethical issue**	**Description of challenge**	**Potential mitigation strategies**
**Consent**	Infeasibility of obtaining explicit informed consent from large numbers of online users. Ambiguity of implied consent via Terms of Service (ToS); users often unaware or uncomfortable.	Restrict analysis to clearly public data. Consider consent at dissemination stage (for quotes). Clearly define research purpose. Ensure transparency about data use. Consult IRB/REC and follow relevant ethical guidelines.
**Privacy**	Blurring of public and private boundaries online. User expectations may differ from platform definitions. Risk of handling sensitive health information without adequate safeguards.	Adhere to data protection laws (e.g., GDPR, HIPAA). Minimize collected data to essentials. Implement strong data security measures. Conduct privacy impact assessments. Respect user privacy settings and platform norms.
**Anonymity/re-identification**	Traditional anonymization often insufficient for online or networked data. Verbatim quotes can easily be traced. Higher risks with geolocation data or rare conditions.	Avoid direct quotes; use paraphrasing or data aggregation. Rigorously remove identifiable information. Secure data storage and control access. Use data intermediaries when appropriate. Assess re-identification risk contextually.
**Bias/fairness**	Models may inherit or amplify biases from unrepresentative data or algorithms, leading to inaccurate conclusions and exacerbating health disparities.	Critically evaluate data representativeness. Use diverse datasets. Apply bias detection and mitigation techniques. Audit model performance across demographic groups. Ensure transparency about data limitations and bias risks. Consider social justice impacts.
**Harm/stigma**	Potential for reputational harm, embarrassment, psychological distress. Risk of stigmatizing individuals or groups based on inferred sentiment or health status. Misuse or misinterpretation of findings.	Carefully weigh benefits vs. risks (principle of nonmaleficence). Avoid labeling individuals. Ensure high data quality and responsible interpretation. Provide support resources when dealing with sensitive topics (e.g., mental health). Anticipate and consider downstream impacts of research dissemination.

#### 3.5.1 Consent, privacy expectations, and the public/private data dichotomy

A central ethical challenge in using online data for public health sentiment analysis involves informed consent. Obtaining explicit consent from potentially millions of users is often impractical. Reviews indicate that reporting of ethical approval and informed consent is frequently inadequate in studies utilizing social media data for health research (8, 16). Relying on platform terms of service as implicit consent is debatable, as many users may be unaware of or uncomfortable with their public data being used for research (16). Standard research ethics emphasize voluntary participation, which is hard to ensure with large-scale data mining.

This issue connects to the blurred line between public and private spaces online. While a post might be technically public, users may still have reasonable expectations of privacy, especially regarding sensitive health topics (11). Ethical practice requires considering user expectations beyond legal definitions (8). Restricting analysis to data explicitly marked as public is a suggested harm reduction strategy. Protecting privacy also demands compliance with data protection laws (e.g., GDPR, HIPAA), implementing security measures, and practicing data minimization–collecting only necessary data (5, 6, 65). As advanced tools like LLMs are integrated into health applications, ensuring patient engagement and transparency regarding their use is also considered a prerequisite (62).

#### 3.5.2 Ensuring anonymity, confidentiality, and mitigating re-identification risks

Maintaining the anonymity and confidentiality of individuals whose online data is analyzed is an important ethical duty (8, 11). However, traditional methods like simply removing names often prove insufficient for online data due to the internet's networked and persistent nature. Verbatim quotes, for instance, can sometimes be traced back to the original source using search engines, potentially revealing the user's identity (16). This re-identification risk can be higher when analyzing discussions involving unique characteristics (like rare diseases) or geo-referenced data (65). Researchers must consider the protection of not only the primary user but also any third parties mentioned in posts.

Several mitigation strategies are necessary. Rigorously stripping identifiable information during data collection is a primary step (8). Implementing secure data storage and access control policies is also important. Instead of reporting individual-level findings or using direct quotes, researchers can use aggregated data analysis (16) or paraphrase sensitive content (16). However, if paraphrasing is used, the methods should be clearly reported (16). Transparency regarding data handling practices helps build trust (65). Careful assessment of re-identification risk within the specific context of the study is necessary (65).

#### 3.5.3 Recognizing and addressing algorithmic bias and fairness

Sentiment analysis algorithms are susceptible to bias (11). Models, including machine learning classifiers and LLMs, can inherit and even amplify societal biases related to characteristics like race, gender, or culture if trained on unrepresentative data or built using biased lexicons (46, 65). Platform demographics themselves can introduce bias if not considered. Studies have demonstrated specific biases in LLMs, such as cultural biases revealed through differing performance on prompts related to distinct cultural contexts (54), or biases influenced by prompt design and label-word choices (38).

The impact of such algorithmic bias in public health can be significant. If biased models produce inaccurate sentiment assessments for certain population groups, the resulting insights may be misleading. This could lead to ineffective or inequitable health interventions, potentially worsening existing health disparities (65).

Addressing this challenge requires proactive measures. Researchers must critically evaluate training data for potential biases and seek for diverse and representative datasets when feasible (65). Utilizing techniques designed to detect and mitigate algorithmic bias during model development and evaluation is important (65). Transparency about data limitations and potential model biases is necessary when reporting findings. Furthermore, ethical considerations should contain broader issues of social justice and potential inequalities stemming from the application of these technologies (11).

#### 3.5.4 Promoting responsible data practices and minimizing harm

When deploying new technologies like LLMs, it is crucial to consider potential harms stemming from their inherent characteristics (detailed in Section 3.1.4 and Section 3.2.5). These include the risk of generating and spreading misinformation (14), and negative impacts that can arise from inappropriate humanization or a lack of operational robustness in sensitive applications (49, 55).

Beyond privacy breaches and bias, using sentiment analysis in public health carries other potential harms. Revealing sensitive information could cause reputational damage or psychological distress (11). Misinterpreting or misusing findings can lead to flawed decisions (65). There is also a risk of analysis contributing to the stigmatization of certain conditions or groups, especially if findings link negative sentiment to specific demographics (11). When deploying new technologies like LLMs, it is crucial to consider potential harms stemming from their inherent characteristics (detailed in Section 3.1.4 and Section 3.2.5). These include the risk of generating and spreading misinformation (14), and negative impacts that can arise from inappropriate humanization or a lack of operational robustness in sensitive applications (49, 55).

Responsible data practices are therefore necessary (65). This includes clearly defining the research purpose, ensuring data quality, and respecting user rights (65). Researchers have a duty of care and must apply the principle of nonmaleficence (“do no harm”), carefully weighing potential research benefits against risks to individuals and communities (11). Specific care should be taken to avoid labeling individuals, particularly regarding sensitive conditions like mental illness. When using advanced tools like LLMs in clinical or public health settings, responsible integration involves developing clear guidelines for use, ensuring appropriate training, and performing safety checks (46, 62). Interestingly, sentiment analysis itself might be used positively to help combat stigma by identifying stigmatizing language online (11). Transparency about methods, data usage, and limitations is fundamental to responsible practice and building public trust (8, 16, 65).

## 4 Future directions

### 4.1 Identifying critical research gaps and unanswered questions

To advance the application of sentiment analysis in public health, several critical research gaps require attention. A primary area concerns the need for robustness, generalizability, and effective domain adaptation. Future research must explore how to develop sentiment analysis models that perform reliably across diverse public health contexts, populations, data sources, and timeframes, as current models often show variability or performance degradation when applied to new domains or specific health language (19, 43, 55). This includes developing effective techniques for adapting large pre-trained models, like LLMs, to health-specific complexities (36, 42). Another significant gap lies in multilingual and low-resource capabilities, as most current research focuses on English, leaving a need for validated methods and resources for a wider range of languages and cultural contexts (35, 54).

Furthermore, the explainability and interpretability (XAI) of complex models remain important unanswered questions. Making the reasoning behind sentiment classifications transparent is important for building trust and enabling actionable insights for public health stakeholders, demanding more research into integrating XAI techniques (41, 52). The development of practical ethical frameworks designed for using online data in public health sentiment analysis is also urgently needed, focusing on balancing public health goals with individual rights concerning consent, privacy, and bias mitigation (8, 11, 16, 65).

Finally, research needs to move beyond identifying correlations toward exploring causality, integration, and impact. Investigating potential causal links between sentiment and health outcomes (56), determining optimal ways to integrate sentiment analysis with traditional public health processes (1, 69), and rigorously evaluating the real-world impact on measurable health outcomes and policy decisions are critical next steps (70, 71). Addressing these interconnected gaps is necessary for sentiment analysis to become a fully realized, reliable, and ethically sound tool in public health.

### 4.2 Emerging trends and future potential

The future of sentiment analysis in public health will likely be shaped by several emerging technological trends. LLMs will continue to be explored for various sentiment analysis tasks, leveraging their few-shot learning capabilities and potential when fine-tuned (15, 36). Future work will likely focus on improving their consistency, reliability, and efficiency for health-specific data (55), applying them to new areas like personalized health insights (50), psychotherapy assistance (39), augmenting clinical workflows (46), and public health interventions (49).

Moving beyond text, multimodal sentiment analysis, which incorporates information from images, videos, or audio, holds potential for a richer understanding of expressed sentiment, as communication is often multimodal (72). The demand for transparency is driving interest in Explainable AI (XAI), aiming to make the reasoning behind sentiment classifications clear, especially for complex models (27, 41). Developing models with better cross-domain and cross-lingual capabilities is another trend, reducing the need for extensive retraining for every new health topic or language (35).

Sentiment analysis is also likely to be increasingly integrated with other AI and ML techniques, such as topic modeling (24, 31, 64, 73) or predictive analytics, to generate more comprehensive public health intelligence (5). This integration supports the potential contribution of sentiment analysis to precision public health, enabling more targeted interventions by identifying sentiment patterns within specific communities or demographic groups. Furthermore, developing robust, validated, and ethical real-time monitoring systems remains a goal, potentially providing valuable early warnings and supporting rapid public health responses (4, 9).

## 5 Discussion

This systematic review provides a broad overview of public health sentiment analysis (SA). We looked into the methods, applications, data sources, common challenges, evaluation techniques, and ethical considerations. Sentiment analysis uses computer methods, primarily from Natural Language Processing (NLP) (9, 67, 74, 75), to understand feelings and opinions within text data, often gathered from online platforms like social media (3, 20, 61). Our review aimed to answer important questions about SA in public health today and its future. Specifically, we focused on the methodologies being used (addressing RQ1), the range of public health applications (RQ2), the data ecosystem and its associated difficulties including ethical aspects (RQ3), and how SA model performance is assessed (RQ4).

Regarding methodologies (RQ1), we observed a clear trend toward more sophisticated techniques (33, 36, 53, 72). Basic lexicon-based methods, which rely on predefined word lists, offer simplicity but often fall short in health contexts due to their insensitivity to domain-specific language and context (19). Traditional machine learning approaches, such as Support Vector Machines (SVM) (76), can learn complex patterns from human-labeled data (24) and perform better than lexicons when suitable data is available. However, creating such specialized health datasets requires significant time and expert knowledge (10, 34). Deep learning models, especially Transformer architectures like BERT (66) and LLMs like GPT (14, 15, 37, 39–41, 43–51, 55, 62, 68), currently offer the best performance in understanding complexities and context (30). These models benefit from pre-training on massive text datasets, reducing the need for extensive task-specific labeling (36, 38, 42). However, they demand considerable computing power, can be difficult to interpret (the “black box” problem) (27), and often need specific adjustments like fine-tuning or careful prompting to work reliably on health-related text (37, 42, 52). Hybrid approaches that combine different methods show potential (72) but require more investigation.

Turning to public health applications (RQ2), our review identified diverse uses for SA. It is frequently employed to monitor public responses to health policies and campaigns, such as those concerning COVID-19 measures (4, 12, 31, 56, 60, 77) or vaccination programs (25, 58), offering faster insights than traditional surveys (12). SA also aids infectious disease surveillance (23) by tracking symptom mentions online (22) and measuring public awareness during outbreaks. Another significant area is monitoring population mental health, analyzing expressions of mood (7, 21, 34, 45, 53, 78) and sometimes attempting to identify suicide risk (44). Furthermore, SA helps understand patient experiences through analyzing online reviews of healthcare services or treatments (24, 29, 40, 43, 63). It also assists in navigating health communication challenges (59), including identifying and combating health misinformation online (14, 42, 66).

Comparing these findings with previous work shows alignment regarding the adoption of advanced methods like deep learning and LLMs (30, 36, 37). The identified applications, such as surveillance and patient experience analysis, are consistent with other reviews (10). However, our synthesis emphasizes the persistent gap between model performance on test datasets and their reliability on real-world public health data (15, 19). Models often perform less effectively in practice (36). Differences compared to earlier findings may stem from the rapid evolution of SA techniques, variations in the primary data sources used [with Twitter being prominent in the studies we reviewed (8, 12, 13, 25, 28, 31, 56, 58, 64, 73)], and differing evaluation standards (19). For instance, while some researchers report high accuracy (29, 30), others find considerable disagreement between different SA tools and human judgments for complex health texts (19, 43).

The evidence synthesized from the 83 included studies varies. While some studies employed robust methodologies, many relied on accessible but potentially biased social media data. We observed variability in data processing, model selection, validation approaches, and reporting transparency across the literature. The heavy reliance on platforms like Twitter and predominantly English-language data restricts the generalizability of many findings. However, specific patterns, like the common applications of SA or the frequent observation of negative sentiment around specific health issues, were consistent across multiple studies, lending some weight to these particular conclusions despite the overall methodological diversity.

We noted significant heterogeneity across studies regarding methods, data sources, and results (related to RQ1 and RQ3). Methodological approaches ranged widely, from lexicon-based (19) to advanced deep learning (27, 30). Data sources were dominated by Twitter (8, 13, 56) but also included other platforms like Reddit (44, 68), online health forums (34), news articles (22), and patient reviews (43, 63). These differences in data and methods naturally lead to varied results. For instance, sentiment patterns depend on the specific population studied [e.g., social media users vs. the general public (8)], the health topic [e.g., COVID-19 vs. mental health (7, 73)], and how sentiment itself was measured (19). This variety means that SA results are often context-specific (11), limiting direct comparisons and broad generalizations.

Our findings have important implications for public health practice and policy (related to RQ2). SA tools can help monitor public opinion in real-time (12), understand reactions to policies (56, 79), evaluate communications (57, 59), support disease tracking (22), assess mental well-being (7, 62), and understand patient feedback (24, 63). These insights can inform policy decisions (2, 69–71, 80), refine interventions (81), target communication (64), and guide resource allocation (40). However, the identified challenges - complex language (38), data quality issues (5, 8), model reliability problems (19), and ethical concerns (8, 11, 16, 55, 65) - require critical interpretation of SA outputs. Public health professionals should ideally use SA insights alongside other data (6) and remain aware of the limitations (8). Relying exclusively on potentially flawed SA results for critical decisions could be harmful (65). Therefore, robust validation (19) and user-friendly tools are needed before SA can be routinely integrated into public health decision-making (1, 82).

This review also identifies critical research gaps (related to RQ1, RQ3, RQ4). There is a strong need for better validation of SA methods, particularly LLMs, on realistic public health data (15, 19, 46). Research must focus on improving model consistency and generalizability across different health topics, populations, data sources, and over time (8). Developing effective methods to adapt models for specific health language (42, 47) and creating resources for more languages (29, 35, 54) are necessary. Enhancing model interpretability using Explainable AI (XAI) is important for trust and accountability (27, 41). Clearer, context-specific ethical guidelines for using online health data are required, addressing consent, privacy, and fairness (8, 11, 16, 65, 83). Future studies should also aim to establish causal links between sentiment and health outcomes, moving beyond simple correlations (56, 84). Finally, research is needed on effectively integrating SA with existing public health systems (1, 76) and rigorously evaluating its real-world impact (70, 85).

Considering alternative views, observed sentiment trends might be influenced by factors other than public opinion, such as changes in social media usage patterns or platform algorithms (32). The heavy reliance on specific data sources like Twitter likely means perspectives from other groups are missed (8). Simplifying sentiment into basic categories may overlook emotional complexity (11, 67). The analytical choices made in primary studies and this review also shape the conclusions. Discrepancies in findings, for example regarding LLM performance (19, 36), highlight the field's rapid changes and the need for careful assessment of results. Potential biases within the included studies require a cautious approach to interpreting the overall evidence.

## 6 Conclusion

This systematic review comprehensively examined sentiment analysis methods and their application within the public health domain. We explored the evolution of techniques from simpler lexicon-based approaches to more advanced machine learning and sophisticated deep learning models, including large language models. Our findings confirm that sentiment analysis is being applied across a wide range of public health areas. These include monitoring public reactions to policies and health campaigns, enhancing infectious disease surveillance, tracking population mental health trends, understanding patient experiences with healthcare, and navigating health communication, particularly in combating misinformation.

Despite the rapid advancements and diverse applications, significant challenges remain. Effectively interpreting complex human language, ensuring the quality and representativeness of data sourced primarily from online platforms, and addressing the scarcity of domain-specific resources like annotated datasets continue to limit the field. Furthermore, the ethical considerations surrounding privacy, consent, bias, and potential harm require careful and ongoing attention. While sentiment analysis offers promising tools for gaining timely public health insights, its practical implementation faces limitations related to model reliability, interpretability, and the need for rigorous validation in real-world settings.

Therefore, the main message from this review is one of cautious optimism. Sentiment analysis holds considerable potential to inform public health practice, policy, and research by providing valuable insights into public attitudes and experiences. However, realizing this potential requires a critical approach. Future progress depends heavily on developing more robust, interpretable, and ethical methodologies. Addressing the existing limitations through further research, developing clear standards for validation and reporting, and promoting responsible innovation are necessary steps. Finally, integrating sentiment analysis effectively into the public health toolkit demands a balanced perspective that acknowledges its strengths and current weaknesses, ensuring that these powerful tools are used reliably and equitably to improve population health.

## Data Availability

The original contributions presented in the study are included in the article/supplementary material, further inquiries can be directed to the corresponding author.
